# International Medical Congress

**Published:** 1881-09

**Authors:** 


### International Medical Congress.

From our London Correspondent.

Of the many letters which will go to American medical journals in regard to professional matters here, I doubt if any will contain more than the most superficial summary of the past week's doings. As soon as the ponderous volume of Transactions is issued, there will doubtless be enough in quantity and quality to satisfy the mental appetite of the most greedy student; perhaps, also, enough to surfeit any one who attempts to digest the whole. Not that the proceedings of the Congress are of an inferior order. Too many precautions were taken by the various committees, and too much interest has been manifested by leading medical men the world over, to make its success at all doubtful. It is only the quantity of matter which the volume of the transactions must contain, which will terrify the reader; but, after all, it will only be in proportion to the number of those present. For there have been 3,210 members inscribed, and the different sections have held 119 meetings, at which there were presented 464 written and 362 verbal communications. This record is not surprising to any one who witnessed the first general meeting at St. James Hall, in Piccadilly. Only members of the Congress and special foreign representatives were admitted, and yet every part of the house was packed, even to the topmost galleries. Medical men had gathered there from all parts of the globe, and many of them the best that their countries could produce. Among the foreigners there were such asVirchow, Esmarch, Langenbeck, Pasteur, Charcot, Flint, Da Costa, and a host of other authorities; while England was represented by her foremost men. The rows of seats flanking the stage were occupied by vice-presidents and members of the various committees, while in front sat the president, Sir James Paget, with the Prince of Wales on one side and the Crown Prince of Germany on the other. After formally organizing, the Congress as usual divided into sections, there being fifteen this time—an illustration of a remark of the president that "if the field of any specialty is narrow, it can be dug deeply."

In each section there was an abundance of papers, as the figures just given would lead one to suppose, and indeed there were occasionally so many on a certain phase of a subject as to monopolize time which might have been devoted to its discussion. And when a debate did take place, it was not always easy to follow the speaker unless the listener was fortunate enough to be a sort of walking polyglot. It frequently happened that one would open the discussion in English, another would reply in German, and still another follow in French. Generally, however, the speaker was asked to repeat briefly in English what he may have said in his own language—and these attempts were sometimes more amusing than instructive. Like children learning to talk, we can all understand the words of another before we can make use of them ourselves. The difficulties which might have resulted in this instance were to a great extent obviated by a precaution of the committee in printing a "brief" of every paper in English, in German, and in French. This list of abstracts alone made rather a cumbersome volume of over seven hundred pages, but it frequently proved exceedingly convenient to our following the different readers or speakers.

Besides the work in the sections we have had the pleasure of listening, each afternoon, to an address by some representative man upon a subject of general interest. Among these the paper of Prof. Virchow is deserving of special mention, and in dealing as he did with "The value of pathological experiment," he took occasion to give the anti-vivisectionists several hearty raps. Dr. Billings, who is in charge of the library at Washington, also quite distinguished himself by his address on "Our Medical Literature," for he has a pleasant way of stating facts, and the frequent applause which followed his quaint and pithy sayings, showed that his effort was thoroughly appreciated. Others, equally well qualified to speak upon the subjects chosen, entertained the Congress at its general meetings—all being physicians except Professor Huxley, who discussed the "Connection of the Biological Sciences with Medicine" in his usual clear and forcible style.

Nor should the social attractions of such a gathering be forgotten; indeed, these London folk offer their hospitalities in such an open-handed fashion as to give constant occupation to those who would accept even a part of the invitations received. The leading medical men of the city have vied with each other in the frequency of their "Luncheons" and other entertainments, while the more elaborate receptions of the Lord Mayor, Earl Granville, Baroness Burdett-Coutts, and similar social leaders, have given glimpses of English life which few of us foreigners could otherwise have obtained. On the whole, the Congress has seemed to prove a success professionally and socially, and the departing guests leave their English friends with only the most delightful impression of their short but enjoyable stay.

